# Generating quantitative binding landscapes through fractional binding selections combined with deep sequencing and data normalization

**DOI:** 10.1038/s41467-019-13895-8

**Published:** 2020-01-15

**Authors:** Michael Heyne, Niv Papo, Julia M. Shifman

**Affiliations:** 1 Department of Biological Chemistry, Hebrew University of Jerusalem, Givat Ram Campus, 91906 Jerusalem, Israel; 2 Avram and Stella Goldstein-Goren Department of Biotechnology Engineering and the National Institute of Biotechnology, Ben-Gurion University of the Negev, P.O.B. 653, 8410501 Beer-Sheva, Israel

**Keywords:** Biochemistry, Biophysics, Biotechnology, Chemical biology, Computational biology and bioinformatics

## Abstract

Quantifying the effects of various mutations on binding free energy is crucial for understanding the evolution of protein-protein interactions and would greatly facilitate protein engineering studies. Yet, measuring changes in binding free energy (ΔΔG_bind_) remains a tedious task that requires expression of each mutant, its purification, and affinity measurements. We developed an attractive approach that allows us to quantify ΔΔG_bind_ for thousands of protein mutants in one experiment. Our protocol combines protein randomization, Yeast Surface Display technology, deep sequencing, and a few experimental ΔΔG_bind_ data points on purified proteins to generate ΔΔG_bind_ values for the remaining numerous mutants of the same protein complex. Using this methodology, we comprehensively map the single-mutant binding landscape of one of the highest-affinity interaction between BPTI and Bovine Trypsin (BT). We show that ΔΔG_bind_ for this interaction could be quantified with high accuracy over the range of 12 kcal mol^−1^ displayed by various BPTI single mutants.

## Abstract (web summary)

Quantifying the effect of mutations on binding free energy is important to understand protein-protein interaction (PPI). Here the authors develop a method based on yeast display and next-generation sequencing to generate quantitative binding landscapes for any PPI regardless of their Kd value.

## Introduction

Protein-protein interactions (PPIs) control virtually all processes in the cell. Mutations at PPI binding interfaces frequently affect free energy of binding (ΔΔ*G*_bind_), sometimes abrogating and sometimes stabilizing the interaction. This change in binding affinity of one PPI could translate into remodeling of the whole PPI network, frequently leading to dysregulation of signal transduction pathways and disease^1,2^. Therefore, understanding how specific mutations in protein complexes affect their binding affinity is extremely important to both basic biology and to biomedical sciences, where inhibition of a particular PPI might help to treat the related disease.

In the recent years, many groups reported computational methods for predicting ΔΔ*G*_bind_ from structure and/or sequence^3–12^. While achieving good predictions on average, these methods frequently give large errors in particular cases, revealing that our comprehension of the precise molecular forces that govern binding affinity in PPIs remains incomplete^13^. Our knowledge in this area could be greatly expanded by acquiring large sets of data for ΔΔ*G*_bind_ values in various PPIs, facilitating progress in computational modeling. Yet, experiments that determine ΔΔ*G*_bind_ remain laborious since they involve DNA manipulation, protein expression and purification in different organisms and binding affinity measurements using different techniques. Thus, experimental data describing mutational effects on binding affinity for each particular PPI remain sparse and sometimes inconsistent between different reported experiments^14^. Furthermore, the majority of mutations reported in the literature are mutations to alanine^14–18^. However, in natural evolution, mutations to Ala are not particularly frequent. Moreover, they rarely lead to binding affinity improvement, which is of interest to most protein engineering studies.

A much more attractive and informative approach would be to explore all possible mutational effects for a particular PPI in a single experiment, thus generating a comprehensive binding landscape for this PPI^19,20^. Such binding landscapes could be used to define evolutionary paths accessible to a particular PPI, to characterize energetic contribution of each position, and to locate frequently sought affinity- and specificity-enhancing mutations^3,21^. First efforts in this direction utilized phage display technology that allows to select binders from a large combinatorial library of protein mutants^20,22,23^. Through several rounds of selection, protein mutants compatible with binding to a particular target are selected. Subsequent sequencing of multiple selected clones allows us to calculate the frequency of each amino acid at each position, providing information on binding hot-spots^24^ and cold-spots^25^. Further studies in this direction utilized yeast surface display (YSD)^26^ for selecting protein binders coupled to next-generation sequencing (NGS) to produce binding landscapes for various PPIs^27^. While YSD enables fast sorting using fluorescently activated cell sorting (FACS), NGS permits more accurate calculation of amino acid frequencies for each of the detected mutants compared to standard Sanger sequencing methodology. The ratio between the amino acid frequency in the selected pool of binders and the same frequency in the initial naive library, referred to as the enrichment value, is calculated for each amino acid at each of the explored position. The enrichment values are then plotted to produce PPI binding landscapes. Variations on this approach have been used to explore a large mutational space and to engineer higher-affinity and higher-specificity protein binders^28–31^.

In spite of great promise of this approach, further studies on different biological systems revealed its potential limitations. While affinity enhancing mutations could be readily identified by this methodology, relatively low correlation (*R* value of 0.5) between the NGS-derived enrichment values and experimental ΔΔG_bind_ values for purified proteins was observed^17^. Additional studies showed that ΔΔG_bind_ could be inferred from the NGS-based enrichment values only in the narrow range of energies from −0.8 to +0.5 kcal mol^−1^ ^32,33^, preventing construction of quantitative binding landscapes for all of the explored mutations with broader range of target affinities. Recent studies suggest that the use of multiple gates for mutant sorting could improve method accuracy and extend its explored affinity range^29,30^. Yet, the methodology still sets a requirement on the concentration of the target protein in the selection experiment; the concentration should be similar to the interaction *K*_d_, thus limiting the application of the approach to only subset of all PPIs with medium affinities. For high-affinity PPIs (*K*_d_ < 10^−10^ M), this condition would imply the usage of very low target protein concentration. At such low concentrations, ligand depletion could occur, meaning that there are not enough target molecules that can bind the ligand molecules on the yeast surface^34^. While this could be in principle overcome by increasing the sample volume and thus the number of target molecules, low pM target concentrations would require sample volumes of several liters, making such experiments impractical. For low-affinity PPIs (*K*_d_ > 10^−5^ M), high concentrations of target protein would be necessary. Yet, many proteins could not be expressed at high concentrations. Moreover, using high protein concentration in YSD experiments could lead to target aggregation and precipitation, thus biasing experimental results.

We introduce an attractive approach that allows us to overcome the abovementioned limitations and to generate quantitative binding landscapes for any PPI, regardless of their *K*_d_ value. Here, we demonstrate the applicability of our approach to a particularly difficult target, a complex between Bovine Trypsin (BT) and its inhibitor BPTI that possesses ultra-high affinity of 10^−14^ M. We show that through our high-throughput NGS-based approach, we can obtain ΔΔ*G*_bind_ values for all BPTI binding interface mutants that correlate extremely well with experimental results on purified proteins over the range of more than 12 kcal mol^−1^ free energy changes. Our method allows us to comprehensively map the binding landscape for this ultra-high affinity interaction, which would be impossible using any alternative technique.

## Results and discussion

### Setting up YSD experiments

**Fig. 1:**
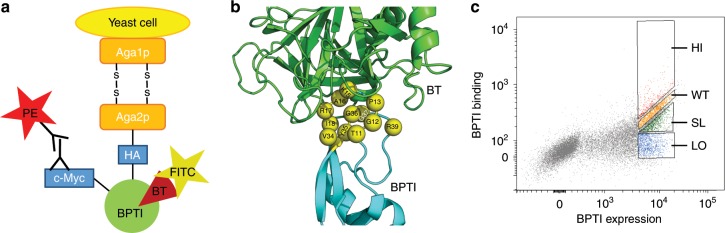
Yeast surface display setup. **a** Yeast surface display construct with BPTI displayed on the surface of yeast cells **b** Construction of the BPTI single mutant library. Structure of the BT/BPTI complex is shown from PDB 3OTJ. BT is colored in green, BPTI is colored in cyan and the BPTI binding interface positions are shown as spheres, labeled and colored in yellow. **c** FACS data showing sorting of four different populations of the BT/BPTI complex. The PE signal monitoring BPTI expression is shown on the x-axis and the FITC signal monitoring binding between fluorescently labeled BT and BPTI variants expressed on the yeast surface is shown on the *y*-axis. The uppermost sorting gate HI represents all mutants with affinity higher than WT. The second uppermost gate WT represents all mutants with an affinity similar to BPTI_WT_. The third gate SL represents all mutants with an affinity slightly lower than WT and the lowest gate LO represents all mutants with an affinity much lower than WT. BT concentration was 5 nM in this experiment.

To demonstrate how our approach could be used to produce quantitative binding landscapes, we first prepared the BPTI/BT complex for YSD experiments. For this purpose, the wild type (WT) BPTI (BPTI_WT_) gene was incorporated into the pCTCON vector, that facilitates BPTI expression on the surface of yeast cells with a C-terminal myc-tag (cMyc) for monitoring protein expression (Fig. 1a). Binding of BT to BPTI mutants was accessed by monitoring fluorescence of the FITC fluorophore conjugated to a biotinylated BT via NeutrAvidin. The assessment of binding of BPTI_WT_ to BT by FACS showed a diagonal narrow distribution, demonstrating that BPTI is well expressed on the surface of yeast cells, is properly folded, and binds to BT (Supplementary Fig. 1).

Next, a combinatorial library was generated containing all single BPTI mutants at positions that are in the direct binding interface with BT excluding two cysteines (C14 and C38) that form a disulfide bond and thus are crucial for BPTI folding. Thus, twelve BPTI positions were randomized to all twenty amino acids with an NNS codon (Fig. 1b). The library of 228 (19 × 12) BPTI single mutants was constructed using the TPCR protocol^35^. The BPTI mutant library was expressed on the surface of yeast cells and incubated with a fluorescently labeled BT at concentration of 5 nM. This concentration of BT was chosen since it was the minimum concentration of BT that resulted in a considerable spread of FACS binding signals from different BPTI mutants to BT (Supplementary Fig. 2).

### Improving accuracy by collecting more data

One of the limitations of previous approaches for binding landscape generation was that ΔΔ*G*_bind_ showed linear dependence on the NGS-enrichment value only in the narrow range of ΔΔ*G*_bind_ values close to zero^33^. The methodology could not previously discriminate between different highly affinity-reducing mutations since such mutations were characterized with the same enrichment values. The same was true for mutations that showed high improvement in affinity. To overcome this limitation and to increase the range of sensitivity for ΔΔ*G*_bind_ predictions, we used multiple affinity gates from which the mutants were collected during the YSD selection experiment. The multiple gates would allow us to collect information for each mutant several times, and each particular mutant would be enriched in at least one affinity gate. In this particular work, we used four affinity gates for mutant collection: higher than WT affinity (HI), WT-like affinity (WT), slightly lower than WT affinity (SL), and strongly lower than WT affinity (LO) (Fig. 1c). The WT affinity gate was set according to the FACS signal produced by BPTI_WT_ binding to BT at same conditions (Supplementary Fig. 1). The cells from each gate were then grown, analyzed for binding to BT (Supplementary Fig. 3) and sequenced with NGS, resulting in 300–900 K reads per each population. In addition, the naive pre-sorted library of BPTI mutants was sequenced.

We further assessed the quality of the NGS data using synonymous mutations as a test. Since some errors in the data could come from errors in the NGS process, especially for sequences detected with low frequency, we tested different cut-off values below which the data on the BPTI mutant would be discarded. Using different cut-off values, we calculated deviations in enrichment values for synonymous mutations expressing the same BPTI variant. Our data shows that at the cutoff value of 100 sequences per BPTI mutant, deviations in enrichment values were negligible (<0.001) (Supplementary Fig. 4). Using this threshold, we were able to detect all 228 BPTI single mutants present in the naive library. No threshold was applied to the sorted populations, since in such a population the low number of sequences was caused by the depletion of that mutant from the population.

**Fig. 2:**
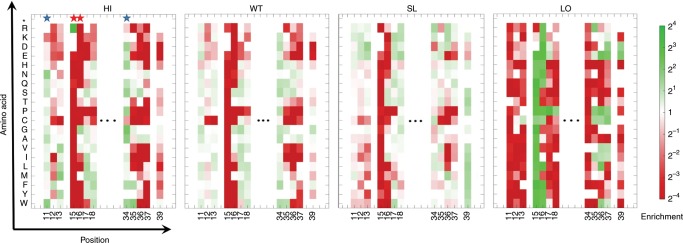
Enrichment values for the BT/BPTI complex. The heat map shows enrichment of each BPTI variant in each of the four affinity groups relative to the naive library as calculated by Eq. (3). The enrichment value varies from high (green) to red (low). Red and blue starts indicate binding hot-spots and tolerant positions, respectively. Source data are provided as a Source Data file.

We thus had in our hands four enrichment values from four affinity gates for each of the 228 BPTI mutants (Fig. 2). Closer examination of the data showed that enrichment values in HI and LO affinity gates exhibited pseudo-symmetry, with highly enriched mutations in the HI gate being highly depleted in the LO gate and vice versa. The enrichment value maps could be used to define binding hot-spots for the BPTI/BT interactions (such as position 15, 16 indicated as red starts on top of Fig. 2) and more tolerant to mutations positions (such as 11 and 34 indicated as blue stars on Fig. 2). However, these maps were not sufficient in determining exact ΔΔ*G*_bind_ values for each of the mutation. In fact, we noticed that some mutations that showed enrichment value of ~1 in the HI gate, that should correspond to the WT-like affinity, were determined to be destabilizing when measured with purified proteins (for example, G12A with experimental ΔΔ*G*_bind_ of +4.35 kcal mol^−1^ ^36–39^). This over-prediction of neutral and affinity-enhancing mutations by our NGS results could be due to the fact that in the YSD selection experiment we used much higher concentration of BT compared to the *K*_d_ of BPTI/BT interaction, shifting the equilibrium towards protein binding even for those BPTI mutants that possessed weaker affinities compared to the WT protein. To overcome this problem, we introduced a normalization strategy described below.

### Normalizing NGS data to get quantitative ΔΔ*G*_bind_ values

To convert the enrichment data from four affinity gates into one ΔΔ*G*_bind_ value, we first collected from literature all available ΔΔ*G*_bind_ experimental data for binding of BPTI single mutants to BT, comprising 29 data points^36–39^. Plotting the experimental ΔΔ*G*_bind_ vs. enrichment values for each of the four affinity gates showed that ΔΔ*G*_bind_ was linearly dependent on the natural log of enrichment values in HI and LO gates (*R*-value of 0.87 for each of the gates, Supplementary Fig. 5). The NGS values from HI and LO gates were denoted further as functions *X*_*1*_ and *X*_*4*_, respectively. ΔΔ*G*_bind_ showed a more complicated two-valued function behavior for WT and SL gates. This was expected since for these gates, the highest enrichment values were observed in the narrow range of ΔΔ*G*_bind_ values but decreased for mutations that showed both higher and lower affinities compared to that narrow range of values. To eliminate this complicated multi-variable behavior and at the same time to utilize the additional information from WT and SL gates, we constructed two additional functions that multiplied enrichment values from HI and SL gates (HI x SL, denoted further as *X*_*2*_) and enrichment values from WT and LO gates (WT x LO, denoted further as *X*_*3*_). These two functions, *X*_*2*_ and *X*_*3*_, were linearly dependent on ΔΔ*G*_bind_. We next used a linear regression to produce the best possible fit of experimental ΔΔ*G*_bind_ values using the linear combination of four functions (*X*_*1*_*, X*_*2*_*, X*_*3*_*, X*_*4*_) arriving to the normalization formula that converts NGS enrichment values into ΔΔG_bind_ for the BPTI/BT complex:

1 $$\Delta \Delta {\boldsymbol{G}}_{{{\mathbf{bind}}}} = - 0.164\,{\boldsymbol{X}}_1 + 0.725\,{\boldsymbol{X}}_2 + 0.364\,{\boldsymbol{X}}_3 + 1.96\,{\boldsymbol{X}}_4 + 5.37$$

**Fig. 3:**
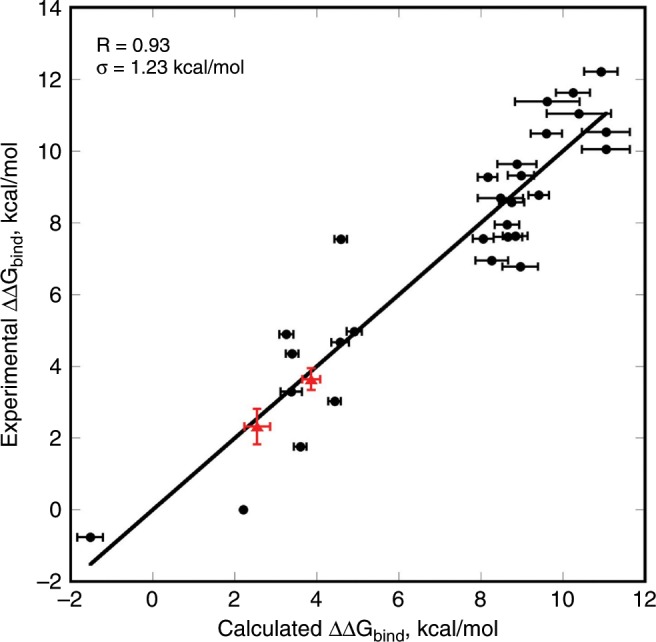
Correlation between the NGS-based vs. experimental ΔΔ*G*_bind_. Experimental ΔΔ*G*_bind_ values were obtained for purified protein variants of BPTI interacting with BT. Black circles show the data taken from the literature. Red triangles show experimental data obtained in this study. Error bars on the X-axis show standard deviations calculated from the bootstrapping of the NGS data. Analysis of the correlation excluding our two data points gives *R*-value of 0.93; *σ* = 1.23 kcal mol^−1^ and *p*-value of 10^−5^ in the two-tailed test. Source data are provided as a Source Data file.

Note that this normalization formula is only valid for this particular NGS experiment and this particular data set of experimental ΔΔ*G*_bind_ values. The formula would change if other experimental conditions such as protein concentration, sorting gates, etc. were to be applied to the YSD experiment or different subset of experimental ΔΔ*G*_bind_ values would be used for normalization. Using 29 data points we were able to predict experimental ΔΔ*G*_bind_ values with very high accuracy over the range of more than 12 kcal mol^−1^ (Fig. 3; *R* = 0.93, *σ* = 1.23 kcal mol^−1^). Analysis of the same data using leave-one-out cross-validation approach, where each data point was predicted without the enrichment information for that particular data point, produced a slightly reduced correlation (*R* = 0.90, *σ* = 1.5 kcal mol^−1^) (Supplementary Fig. 6a). Interestingly, using the enrichment values from only two gates (HI and LO), we were able to predict experimental ΔΔ*G*_bind_ values with only slightly worse accuracy compared to when we used the information from all four gates (Supplementary Fig. 6b). To further access the validity of our approach, we expressed and purified two additional BPTI mutants (R39I and T11N) and measured their ΔΔ*G*_bind_ to BT (Supplementary Fig. 7). Figure 3 shows that our experimental ΔΔ*G*_bind_ values for these two mutants were in very good agreement with predictions from the NGS data analysis.

We used the obtained normalization formula to convert the enrichment values to ΔΔ*G*_bind_ values for all the remaining BPTI mutants in the library. We have further estimated the uncertainty of our ΔΔ*G*_bind_ predictions by applying bootstrapping procedure to the NGS data^40^ and further propagating the error to calculate the 95% confidence interval for each particular ΔΔ*G*_bind_ prediction (see Methods for details).

**Fig. 4:**
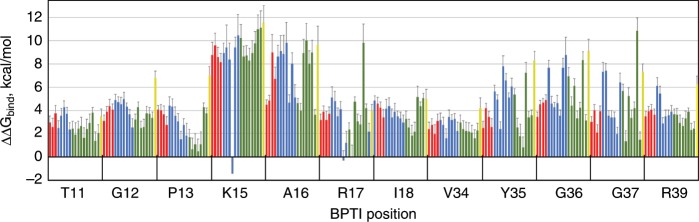
Binding landscape of the BPTI/BT interaction. Changes in ΔΔ*G*_bind_ for all single mutants of BPTI interacting with BT. Each bar represents a mutation to one amino acid including polar amino acids (red), hydrophobic amino acids (green), charged amino acids (blue) and Cys (yellow). *X*-axis shows WT residue followed by position on BPTI. 95% confidence interval for each ΔΔ*G*_bind_ value is shown as calculated from the bootstrapping of the NGS data (see the “Methods” section). Source data are provided as a Source Data file.

Figure 4 shows the ΔΔ*G*_bind_ values for all single BPTI mutants at 12 binding interface positions interacting with BT, producing a quantitative binding landscape for this PPI. As can be seen, the majority of the mutations at all positions in this PPI are highly destabilizing, producing destabilization as high as almost 12 kcal mol^−1^. The most non-tolerant to substitution positions are 15 and 16 that lie in the core of the binding interface (Fig. 1b). However, at position 15, one mutation, K15R, was determined to substantially stabilize the complex, in agreement with experimental results on purified proteins^38^. Figure 4 also shows that the same type of mutations (e.g., hydrophobic or polar) frequently produce similar changes in ΔΔ*G*_bind_ for the same position. We thus established that the BPTI/BT complex with the *K*_d_ of 10^−14^ M is highly optimized by nature, with most single mutations in BPTI leading to high destabilization of the PPI, and very few neutral and affinity-enhancing mutations.

In summary, we report a powerful approach that allows us to produce quantitative binding landscapes based on binding selections into several affinity gates, NGS of the selected mutants, and normalization procedure using a small data set of experimentally determined ΔΔ*G*_bind_ values. Very recently, a similar direction has been taken by Keating and colleagues to design mutations and improve affinity in peptide/protein interactions^41^. The authors also used multiple affinity gates to sort their peptide mutants, but unlike our study, they normalized their NGS data using the apparent binding affinity of peptide mutants on yeast to predict ΔΔ*G*_bind_ for various peptide mutants. The reported correlations between the NGS data and the apparent affinities vary from 0.6 to 0.92 depending on the system over the ΔΔ*G*_bind_ range of 5 kcal mol^−1^. Additionally, Kinney and colleagues, reported a multigate-based strategy that uses several target concentrations in the sorting experiment to produce NGS-derived titration curves for each mutant. This study also achieves high correlation with apparent binding affinities on yeast (*R*-value ranging from 0.82 to 0.89) for the antibody/fluorescein interaction^31^. Similar thinking shown in different independent studies attest to attractiveness of the multi-gate approach for binding landscape mapping. The advantage of our approach includes a simpler normalization procedure using actual in vitro affinity measurements and the possibility to explore PPIs at the limits of the *K*_d_ spectra. We demonstrate superior correlations between ΔΔ*G*_bind_ predictions and actual in vitro measurements over a much larger range compared to all previously reported approaches.

To achieve good prediction accuracy, the experimental data points used for normalization should show large spread in ΔΔ*G*_bind_ values, including both affinity-enhancing and affinity-reducing mutations. The multiple-gate sorting strategy proved to be advantageous over the one-gate sorting strategy for a number of reasons. First, multiple-gate sorting strategy improves the accuracy of predictions by averaging the errors associated with the YSD setup. While the use of only one gate (HI or LO) for ΔΔ*G*_bind_ predictions allows us to achieve good correlation with experiment (*R* = 0.87; *σ* = 1.66 kcal mol^−1^), incorporating additional information from LO gate improves correlation (*R* = 0.89; *σ* = 1.54 kcal mol^−1^) and incorporation of the data from all four gates results in the highest accuracy of prediction (*R* = 0.93; *σ* = 1.23 kcal mol^−1^). Second, we observe that each particular affinity gate is more sensitive in a certain range of ΔΔ*G*_bind_ values. For example, the LO gate in our case proved to be more accurate for predictions of close-to-zero ΔΔ*G*_bind_ values, while the HI gate was more sensitive in the range of very large positive ΔΔ*G*_bind_ values (Fig. S5). Third, the use of multiple gates would become particularly beneficial when mapping affinity changes for a very large number of mutants, that could not be sampled with high frequency by NGS. That is, if a mutant has not been sequenced in some of the affinity gates, we would still be able to make ΔΔ*G*_bind_ predictions based on the information from the gates where this mutant was sequenced. In our experiment, the separation of the mutants to neighboring affinity gates was not perfect; improving gate separation by leaving larger spaces between the gates could further improve the method accuracy. More affinity gates could be also used in future experiments, although the normalization would require a larger set of experimental data for a larger number of gates—at least five data points per each parameter should be used in the normalization function to avoid overfitting.

Our protocol greatly reduces the experimental time for mapping of the binding landscapes. While expression, purification, and binding measurements for hundreds and thousands protein mutants could take months to years, our protocol requires to perform such laborious experiments for only a small subset of mutants and to construct the full binding landscape based on this partial data. Our methodology could be applied to study the evolutionary paths of any PPI regardless of its *K*_*d*_ value and to compare binding landscapes of various PPIs. The approach could be easily extended to studies of double and higher-order mutational steps, providing more comprehensive information on PPI evolution and facilitating future modeling and protein engineering studies. The application of our approach to multiple protein complexes and comparison of different binding landscapes would bring invaluable information about protein evolution. In addition, our approach could be used in various drug design efforts, where antibodies are engineered and affinity matured for interaction with their target.

## Methods

### BPTI library construction

The BPTI_WT_ was generated by PCR using overlapping oligonucleotides (see Supplementary Note 1). The final PCR assembled fragment was gel-purified and cloned into pCTCON vector via transformation by electroporation of *S. cerevisiae* yeast cells (Strain: EBY100 from ATCC, Catalog number MYA-4941) and homologous recombination with the linearized vector (digested with *Nhe*I and *Bam*HI)^42^. Twelve BPTI libraries were constructed from the BPTI_WT_ gene by randomizing each of the binding interface positions with an NNS codon utilizing a TPCR protocol^35^ with one forward and one backward primer (see Supplementary Note 2). The PCR product was treated with DpnI to remove any parental plasmid, cleaned up with magnetic beads, transformed into *E. coli* and selected colonies were sequenced to confirm the successful generation and transformation of the BPTI library. The DNA containing each BPTI library was extracted and all the sublibraries were pooled together and balanced by their DNA concentration. Then, the pooled naive library of BPTI single mutants was transferred into yeast using 20 transformations resulting into 60,000–70,000 colonies for the complete library.

### YSD sorting experiments

Yeast cells displaying the BPTI library or the BPTI_WT_ with a cMyc-tag at the C-terminus on the YSD were grown in SDCAA selective medium and induced for BPTI protein expression with a galactose-containing SGCAA medium as previously described^43^. BPTI expression and binding to individual proteases were detected by incubating approximately 1 × 10^6^ yeast cells with a 1:50 dilution of mouse anti-cMyc antibody (9E10, Abcam, Catalog number: AB-ab32, Cambridge, UK) in 1× Phosphate buffered saline (PBS) supplemented with 1% bovine serum albumin (BSA, Thermo Fisher Scientific, Waltham, MA) for 1 h at room temperature, washed with ice-cold 1xPBS and then incubated with different concentrations of biotinylated BT (biotin and biotinylation protocol from Thermo Fisher Scientific, Waltham, MA) in 1×PBS with 1% BSA for 1 h at room temperature. Thereafter, cells were washed with ice-cold 1×PBS, followed by incubation with a 1:50 dilution of phycoerythrin (PE)-conjugated anti mouse secondary antibody (Sigma-Aldrich, St. Louis, MO, Catalog number: P9670) and 1:800 dilution of NeutrAvidin (Thermo Fisher Scientific, Waltham, MA, Catalog Number: A2662) conjugated with FITC in 1×PBS with 1% BSA for 20 min on ice. Finally, the cells were washed with ice-cold PBS, and the fluorescence intensity was analyzed by dual-color flow cytometry (Accuri C6, BD Biosciences). The yeast cells were next sorted into four populations by FACSAria (BD Biosciences, San Jose, CA) including HI, WT, SL, and LO populations. Sorted cells were then grown in a selective medium, the plasmidic DNA was extracted for each of the sorted population and the naive library and submitted to NGS by MiSeq, Illumina (service provided by Hylabs, Rechovot, IL).

### NGS analysis

The paired-end reads from the NGS experiments were merged^44^ and their quality scores were calculated in the FastQC tool (https://www.bioinformatics.babraham.ac.uk/projects/fastqc/). In the Matlab script, the sequences were aligned, and sequences containing more than one mutation were filtered out. The number of each remaining BPTI mutation *i* in position *j* was counted in the sorted and the naive populations and its frequency *f*^*i,j*^ in the libraries was calculated (Eq. 2). Using the frequency of the mutant in one of the sorted populations and the naive population, the enrichment *e*^*i,j*^ of each BPTI mutant was calculated (Eq. 3).

2 $$f^{i,j} = \frac{{\mathrm{count}^{i,j}}}{{\mathrm{count}_{\mathrm{total}}}}$$

3 $$e^{i,j} = {\mathrm{ln}}\left( {\frac{{\left( {f^{i,j}} \right)_{\mathrm{sorted}}}}{{\left( {f^{i,j}} \right)_{\mathrm{naive}}}}} \right)$$

To estimate the uncertainty in BPTI mutant frequencies we applied a bootstrapping method to the NGS data for all sorted gates and the naive library as described in ref. ^40^. Briefly, the original NGS data was used to randomly draw sequences to obtain a resampling data set of the same size and to calculate the frequency of each BPTI mutant in each population. The resampling process was repeated 1000 times and the average frequency and the standard deviation was calculated from 1000 resampling data sets for each BPTI mutant in each sorting gate and in the naive library. The error was propagated into Eqs. (2) and (3) to calculate the error in enrichment values:

4 $$\partial e^{i,j} = \frac{{\left( {f^{i,j}} \right)_{\mathrm{naive}}}}{{\left( {f^{i,j}} \right)_{\mathrm{sorted}}}}\sqrt {\left[ {\left. {\partial \left( {\left( {f^{i,j}} \right)_{\mathrm{sorted}}} \right)\frac{1}{{\left( {f^{i,j}} \right)_{\mathrm{naive}}}}} \right)} \right]^2 + \left[ { - \partial \left( {\left( {f^{i,j}} \right)_{\mathrm{naive}}} \right)\frac{{\left( {f^{i,j}} \right)_{\mathrm{sorted}}}}{{\left( {f^{i,j}} \right)_{\mathrm{naive}}^2}}} \right]^2}$$

All available experimental data on ΔΔ*G*_bind_ for the BPTI/BT complex was used to obtain the best normalization formula for converting enrichment values from four sorted populations into ΔΔ*G*_bind_ values. To this end, we used a linear regression model function in Mathematica (Wolfram Research) with five parameters (*Y* = a*X*_*1*_ + *bX*_*2*_ + *cX*_*3*_ + *dX*_*4*_ + *f*). The parameters *a*, *b*, *c*, *d*, *f* were optimized using 29 experimental data points as values of Y and the set of *X*_*1*_*, X*_*2*_*, X*_*3*_*, X*_*4*_ values. The obtained normalization formula was used to calculate ΔΔ*G*_bind_ values for all the remaining single BPTI mutants sampled in the NGS experiment, for which no ΔΔ*G*_bind_ values were previously measured. To calculate the uncertainties in ΔΔG_bind_ predictions we propagated the errors in enrichment values into the normalization formula (Eq. 1). The standard deviation of ΔΔ*G*_bind_ predictions for each BPTI mutant was calculated according to the formula:

5 $$	\sigma =\\ 	\sqrt {(a\,\partial X_1)^2 + (b\,\partial X_2)^2 + (c\,\partial X_3)^2 + (d\,\partial X_4)^2 + (X_1\,\partial a)^2 + (X_2\,\partial b)^2 + (X_3\,\partial c)^2 + (X_4\,\partial d)^2 + \partial f^2}$$

where *a*, *b*, *c*, *d* are the coefficients in front of *X*_*1*_, *X*_*2*_, *X*_*3*_, and *X*_*4*_ in Eq. (1), respectively; ∂*X*_1_, ∂*X*_2_, ∂*X*_3_, ∂*X*_4_ are the standard deviations on these variable obtained from the bootstrapping analysis of the NGS data and ∂*a*, ∂*b*, ∂*c*, ∂*d*, ∂*f* are the standard deviations of these coefficients obtained from the leave-one-out analysis. 95% confidence level was calculated assuming a normal distribution as CI = 1.96*σ.

### BPTI mutant expression and purification

The BPTI_WT_ sequence was cloned into a pPIC9K vector and desired mutation was introduced by the TPCR protocol^35^. The mutants were expressed in *P. pastoris* (*GS115* strain, ATCC^®^ 20864) and purified by nickel affinity chromatography, followed by size-exclusion chromatography, as described in previous work^43^. The correct DNA sequence of each produced protein was confirmed by extracting the plasmidic DNA from *P. pastoris* and sequencing. Protein purity was validated by SDS-PAGE on a 20% polyacrylamide gel, and the mass was confirmed with mass spectrometry.

### Ki measurements

Binding affinity between the BPTI mutants and BT was measured using the enzyme activity assays in the absence and in the presence of the BPTI inhibitor (adapted from ref. ^39^). BT and its substrate benzyloxycarbonyl-Gly-Pro-Arg-*p*-nitroanilide (Z-GPR-pNA) that absorbs at 410 nM upon digestion, were purchased from Sigma-Aldrich, St. Louis, MO. Ten samples of a BPTI mutant at different concentrations were prepared and incubated with the substrate Z-GPR-pNA (at a final concentration of 130 μM). An additional sample was made with no BPTI mutant added. BT was added to each sample at 25 pM final concentration. The reaction was allowed to proceed at 25 C and monitored at 410 nM for 16 h. After several hours, the equilibrium was reached and the slope would become linear. Only this linear portion of the data was used for analysis. The data was fit to the following equation to obtain the apparent inhibition constant $K_i^{\mathrm{app}}$:

6 $$\frac{{V_i}}{{V_0}} = 1 - \frac{{\left( {\left[ E \right] + \left[ I \right] + K_i^{\mathrm{app}}} \right) - \sqrt {\left( {\left[ E \right] + \left[ I \right] + K_i^{\mathrm{app}}} \right)^2 - 4[E][I]} }}{{2[E]}}$$

where *V*_*i*_ is enzyme velocity in the presence of inhibitor; *V*_0_ is enzyme velocity in the absence of inhibitor; [*E*] is enzyme concentration; [*I*] is inhibitor concentration. *K*_*i*_ was further determined from the following equation:

7 $$K_i^{\mathrm{app}} = K_i\left( {1 + \frac{{\left[ S \right]}}{{K_m}}} \right)$$

where [*S*] is substrate concentration; *K*_*m*_ is Michaelis-Menten constant that was measured to be 25 μM. Finally, ΔΔ*G*_bind_ was calculated according to:

8 $$\Delta \Delta G_{\mathrm{bind}} = - kT\mathrm{Ln}\left( {\frac{{K_i^{\mathrm{WT}}}}{{K_i^{\mathrm{MUT}}}}} \right)$$

### Reporting summary

Further information on research design is available in the Nature Research Reporting Summary linked to this article.

## Supplementary information

Supplementary Information

Reporting Summary

## Data Availability

All data is available from the authors. The source data underlying Figs. 2–4 and Supplementary Figs. 5–7 are provided as a Source Data file.

## Source data

Source Data
