# Supplementary material for: Generating quantitative binding landscapes through fractional binding selections combined with deep sequencing and data normalization
Source: Nat Commun. 2020 Jan 15;11:297. doi: 10.1038/s41467-019-13895-8 (PMC6962383; doi:10.1038/s41467-019-13895-8)
Supplement: Supplementary file 1 — Supplementary Information [file 41467_2019_13895_MOESM1_ESM.pdf]

Supplementary Information for the manuscript:

Generating quantitative binding landscapes through fractional binding selections combined with deep sequencing and data normalization

Heyne et al

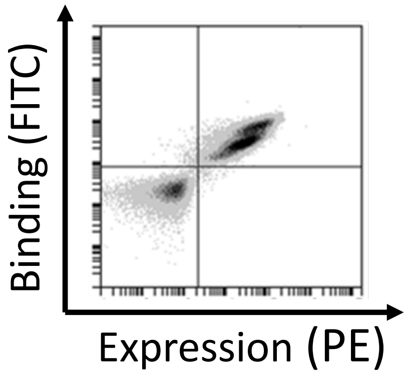

Supplementary Figure 1: FACS data for wild type BPTI. FACS data of $BPTI_{WT}$ binding to 5 nM BT. BPTI Expression was monitored by PE fluorescence while binding to BT was monitored by FITC conjugated to biotinylated BT.

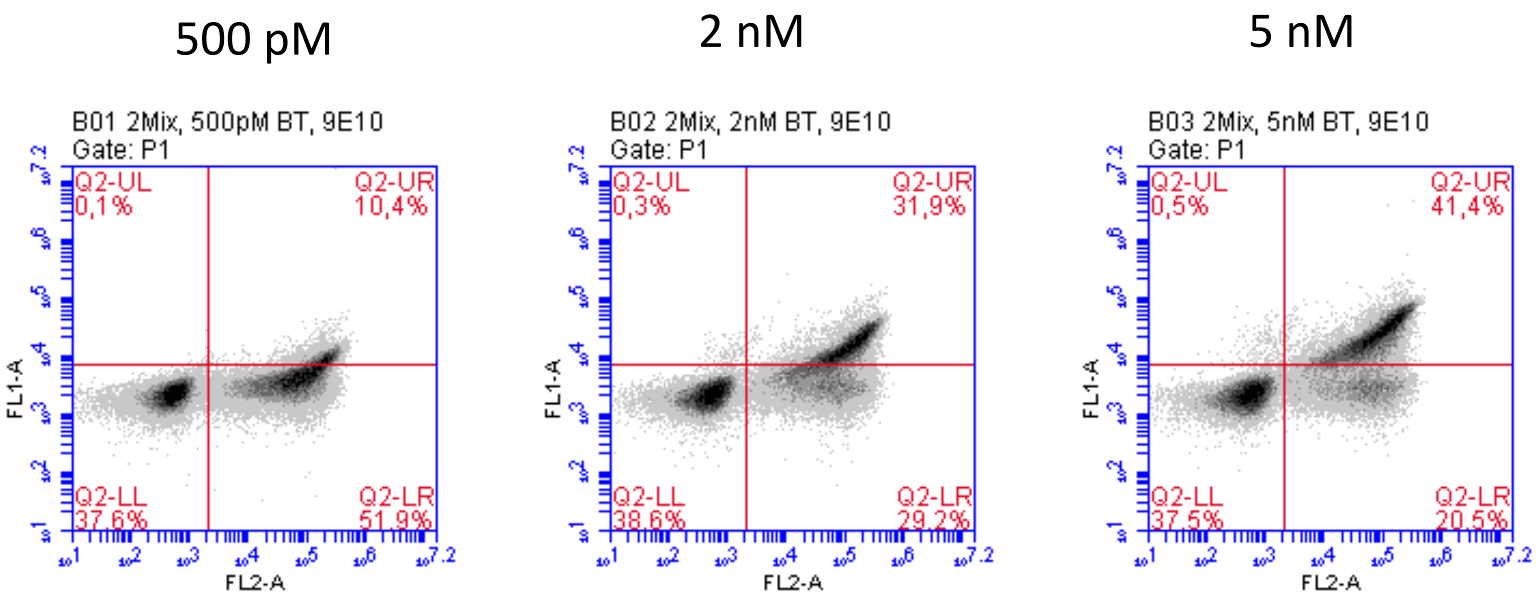

Supplementary Figure 2: Optimizing BT concentration for YSD selections.

The library of BPTI mutants was incubated with different concentrations of BT (500 pM, 2nM, and 5 nM) and the FACS signal was recorded. 5nM concentration was picked for the sorting experiment due to the large spread in binding signals from different BPTI mutants that allows to perform sorting into 4 different gates.

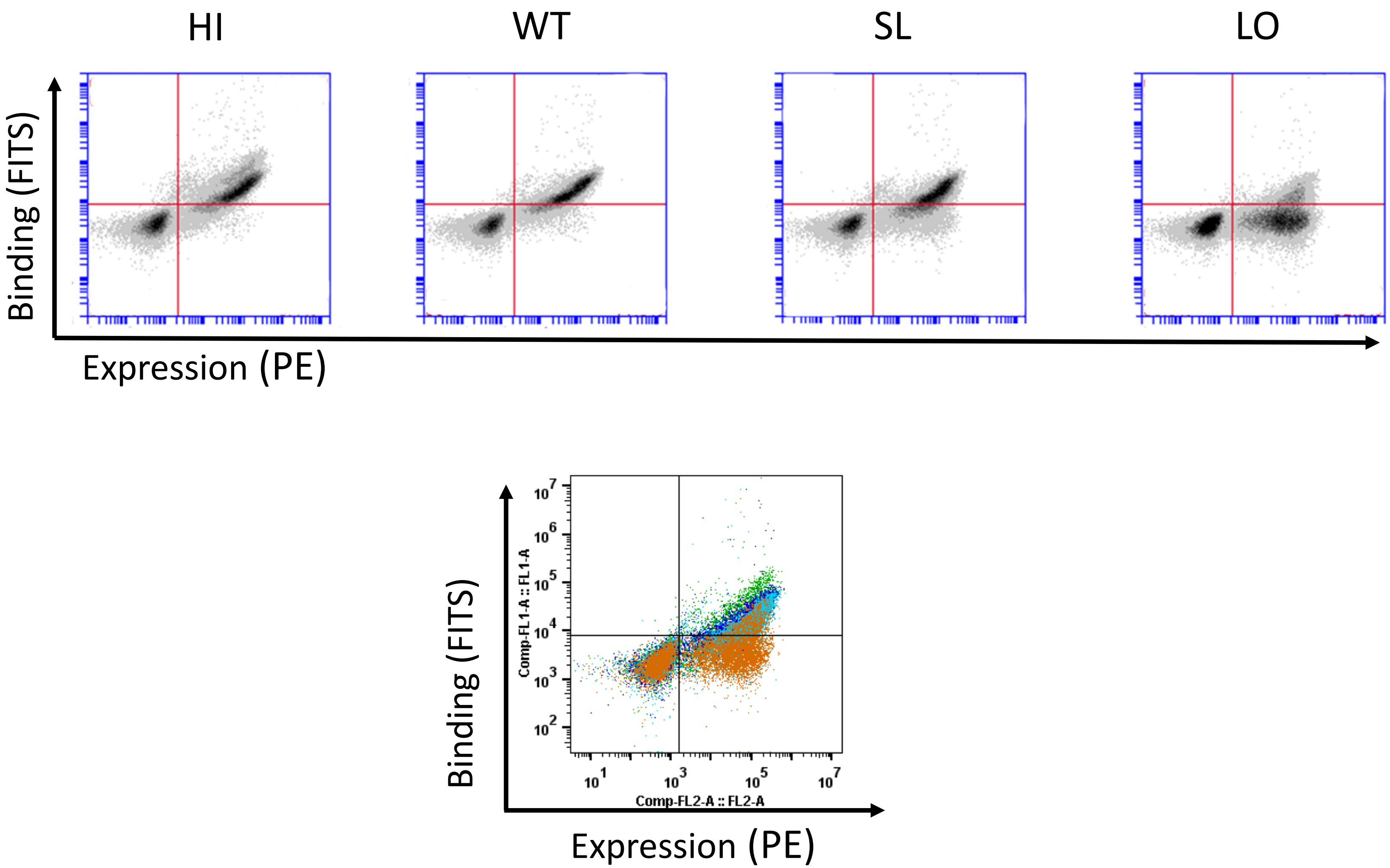

Supplementary Figure 3: Sorting into 4 affinity gates. Top: Separation of BPTI mutant clones into four affinity windows: HI, WT, SL, and LO. The FACS analysis after yeast cells were sorted into 4 gates as shown on Figure 1C and each sorted population of cells was re-grown. Bottom: The data from different gates was overlaid with cells in LO gate (orange), SL gate (cyan), WT (blue) and HI (green).

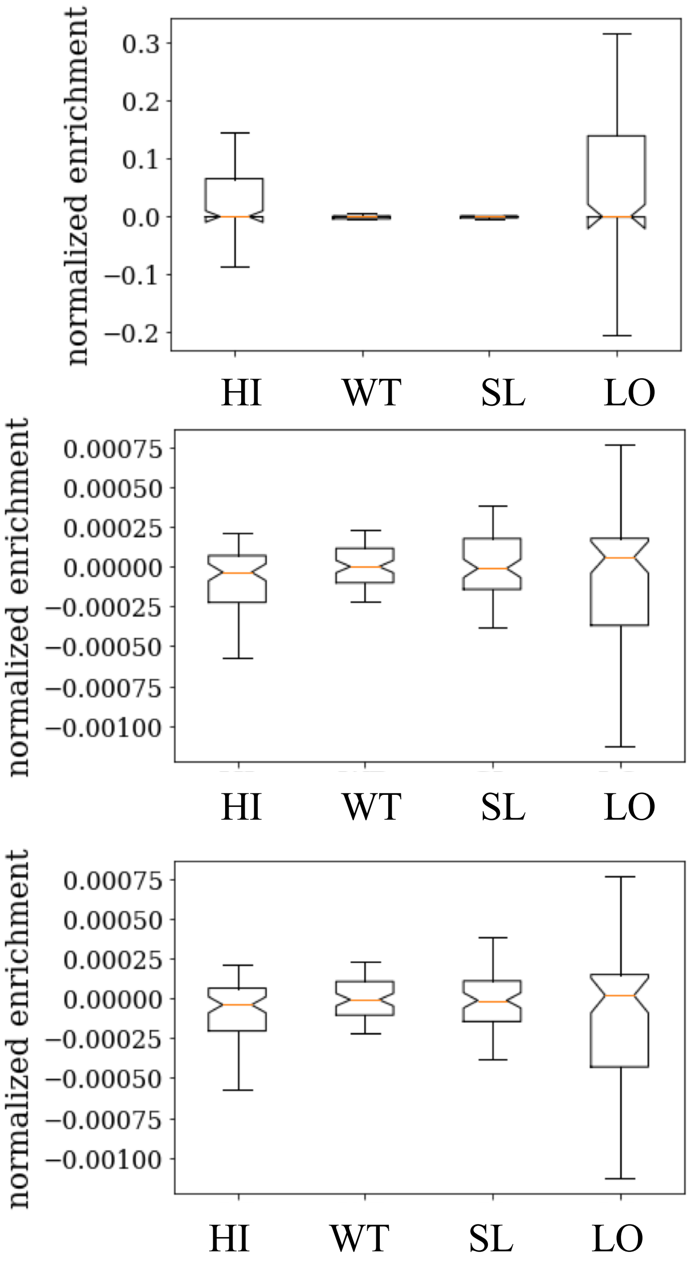

Supplementary Figure 4: Synonymous mutations. Boxplots showing the distribution of the log2 of enrichment of synonymous mutations normalized to the enrichment of the DNA WT sequence for three different thresholds: A) 10, B) 100 and C) 250. Outliers with > 2σ deviations from the average were removed for better visualization.

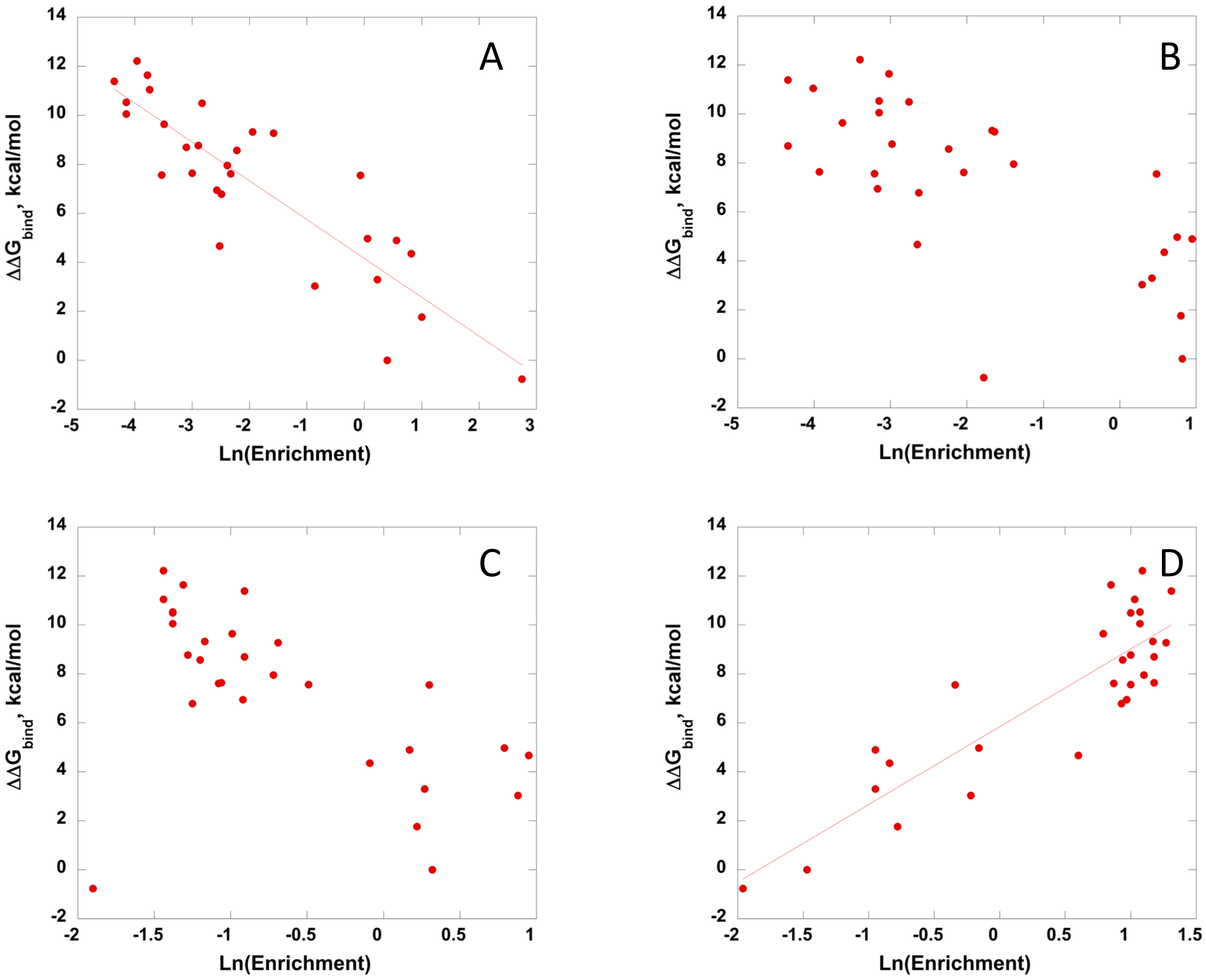

Supplementary Figure 5: Dependence of experimental ΔΔGbind on enrichment value from each gate: (A) HI; (B) WT; (C) SL; (D) LO. R-value is 0.87 and for both HI and LO populations. The source data is available as the Source Data file.

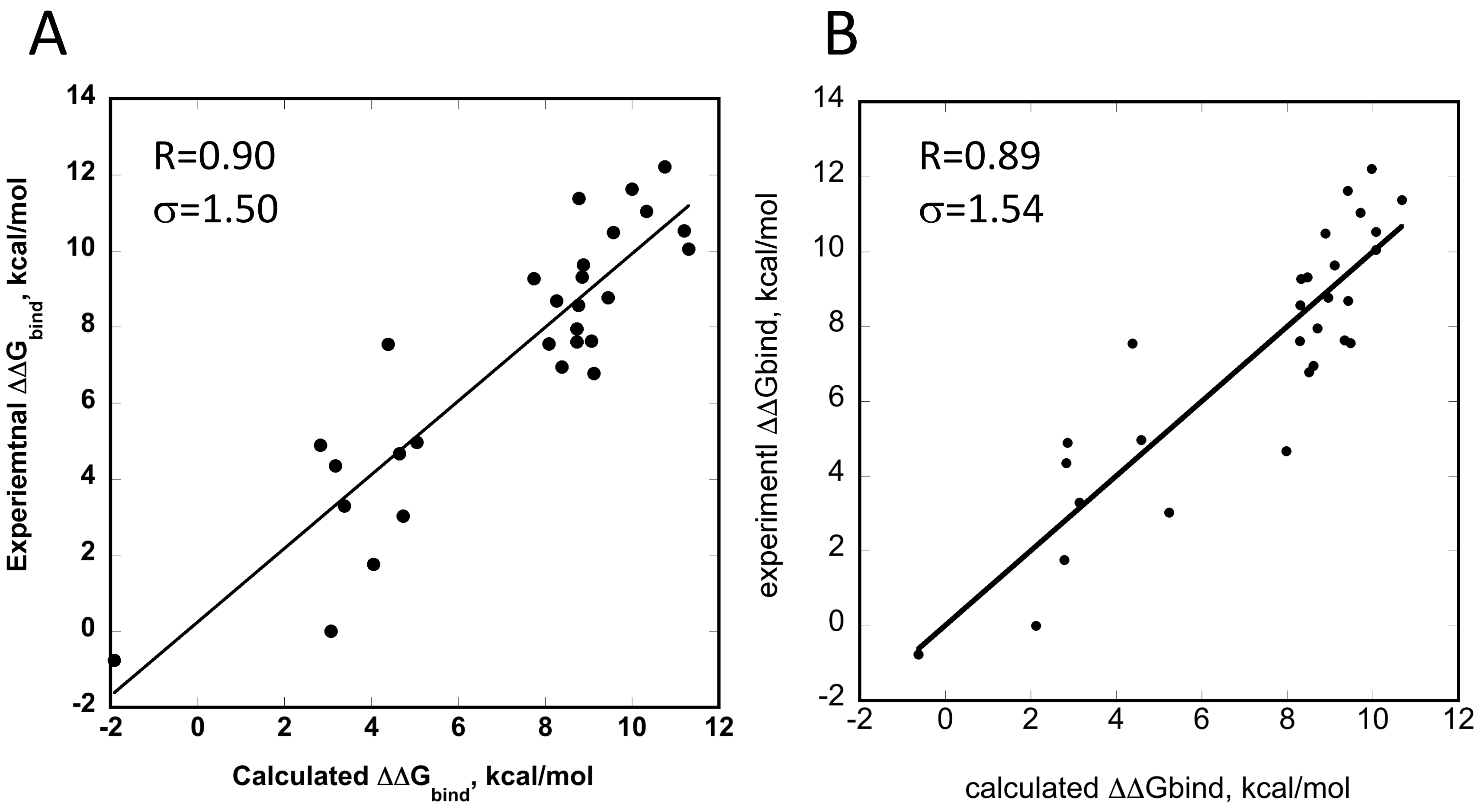

Supplementary Figure 6: Correlation between the calculated $\Delta\Delta G_{\mathrm{bind}}$ using the enrichment values from the NGS results and the $\Delta\Delta G_{\mathrm{bind}}$ measurements for purified protein variants of BPTI interacting with BT. (A) Leave-one-out cross-validation was used to produce this plot, thus each point was predicted without taking into account the information on this particular point. Normalization formula was based on 4 affinity gates as shown on eq. 1. (B) Normalization formula was based on only 2 affinity gates, HI and LO and was obtained using linear regression analysis with 3 parameters. Predictions were done from all data points, that is no data points were left out. The data for this Figure could be found in the source data file.

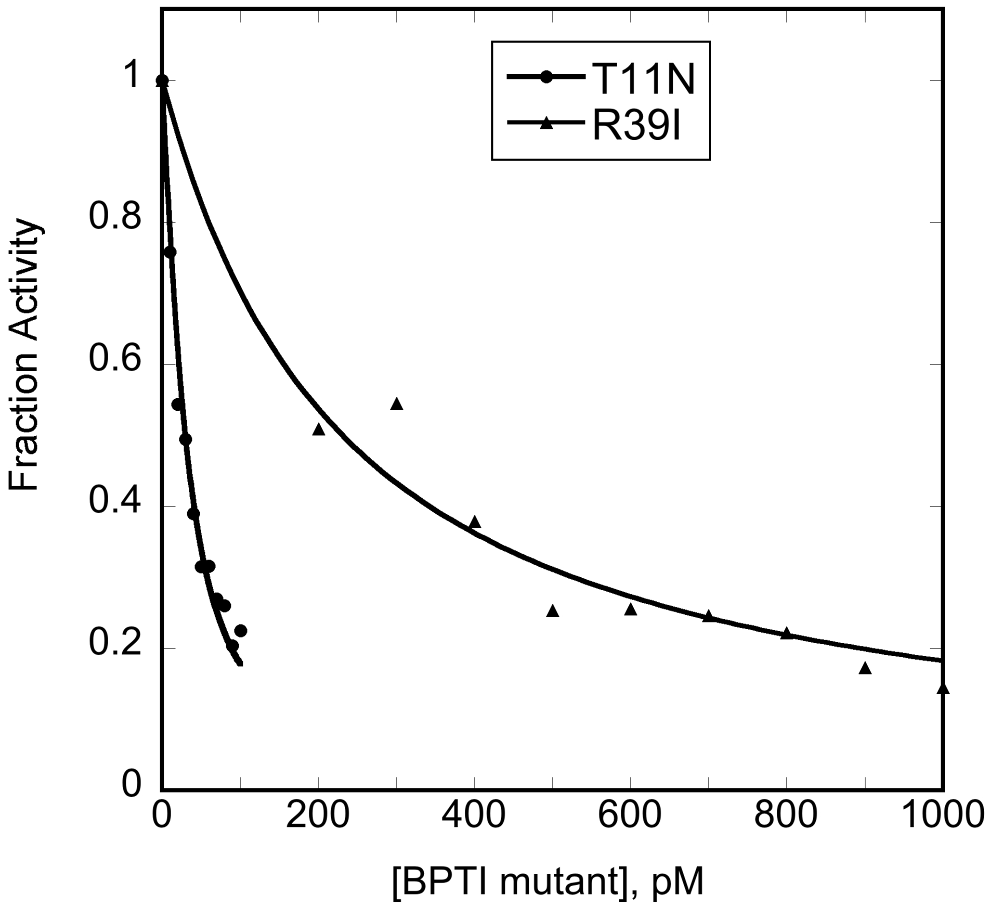

Supplementary Figure 7: Enzyme activity assays for BT showing inhibition by the two selected BPTI mutants T11N and R39I. The experiments were performed as described in the Methods and the data was fit to eq. 5 and 6 to determine *Ki* values. 3 independent experiments were performed to compute average *Ki* ± S.D. $\Delta\Delta G_{bind}$ was calculated assuming Ki of 8 x 10^{-14} M for the WT BPTI as previously reported. The Ki was fitted to be 4 ± 2 and 38 ± 13 pM for T11N and R39I, respectively. The source data is available as the Source Data file.

Supplementary Note 1

WT BPTI gene design

BPTIwt (UniPROT-ID: P00974, 36-93):

NheIRPDFCLEPPY**TGPC****KARI**IRYFYNAKAGLCQTF**VYGGC****R**AKRNNFKSAEDCMRTCGGABamHI

BPTIwt+ Homo:

GGTGGTTCTGGTGGTGGTGGTTCTGGTGGTGGTGGTTCTGCTAGCAGACCAGATTTCTGCCTTGAACCACCATACACGGGTCCCTGCAAGGCAAGGATAATTAGGTA
CTTTTACAATGCGAAAGCAGGTCTGTGCCAGACCTTCGTGTATGGGGGTTGCAGAGCCAAGAGAAATAACTTCAAGTCAGCTGAAGACTGCATGAGGACGTGCGGA
GGCGCTGGATCCTTGCCAGATAAACCATTGGCTTTCCAAGATCCATCTGAACAAAAGCTTATTTCTGAAGAGGACTTGTAATAGCTC

Primers for assembly PCR of the WT BPTI gene

(1) BPTI_F2 (fwd) GGTGGTTCTGGTGGTGGTGGTTCTGGTGGTGGTGGTTCTGCTAGCAGACCAGATTTCTGCCTTGAACC

(2) BPTI_F1 (fwd) GACCAGATTTCTGCCTTGAACCACCATACACGGGTCCCTGCAAGGCAAGGATAATTAGGTACTTTTACAATGCGAAAGCAGGTCTGTG

(3) BPTI_TEMP_F (fwd) CAATGCGAAAGCAGGTCTGTGCCAGACCTTCGTGTATGGGGGTTGCAGAGCCAAGAGAAATAACTTCAAGTCAGC

(4) BPTI_B1 (rev) GCCAATGGTTTATCTGGCAAGGATCCAGCGCCTCCGCACGTCCTCATGCAGTCTTCAGCTGACTTGAAGTTATTTCTCTTGGC

(5) BPTI_B2 (rev) GAGCTATTACAAGTCCTCTTCAGAAATAAGCTTTTGTTCAGATGGATCTTGGAAAGCCAATGGTTTATCTGGCAAGG

Primers for NGS

Seq1-FWD ACACTGACGACATGGTTCTACAACCCATACGACGTTCCAG (61°C)

Seq1-REV TACGGTAGCAGAGACTTGGTCTACGTCCTCATGCAGTCTT (58°C)

Supplementary Note 2

Primers for incorporating single mutations

GGTGGTTCTGGTGGTGGTGGTTCTGGTGGTGGTGGTTCTGCTAGCAGACCAGATTTCTGCCTTGAA

11 12 13 15 16 17 18

CCACCATACACGGGTCCCTGCAAGGCAAGGATAATTAGGTACTTTTACAATGCGAAAGCAGGTCTGT

34 35 36 37 39

GCCAGACCTTCGTGTATGGGGGTTGCAGAGCCAAGAGAAATAACTTCAAGTCAGCTGAAGACTGCAT

GAGGACGTGCGGAGGCGCTGGATCCTTGCCAGATAAACCATTGGCTTTCCAAGATCCATCTGAACA

AAAGCTTATTTCTGAAGAGGACTTGTAATAGCTC

Reverse Complement:

GAGCTATTACAAGTCCTCTTCAGAAATAAGCTTTTGTTCAGATGGATCTTGGAAAGCCAATGGTTTATCTGGCAAG
GATCCAGCGCCTCCGCACGTCCTCATGCAGTCTTCAGCTGACTTGAAGTTATTTCTCTTGGCTCTGCAACCCCCATA
CACGAAGGTCTGGCACAGACCTGCTTTCGCATTGTAAAAGTACCTAATTATCCTTGCCTTGCAGGGACCCGTGTAT
GGTGGTTCAAGGCAGAAATCTGGTCTGCTAGCAGAACCACCACCACCAGAACCACCACCACCAGAACCACC

Legend

Green/Red — Restriction site

Blue — Positions intended to mutate

BPTI_TPCR_ FWD (forward) GATTTCTGCCTTGAACCACCATACAC (26 bases, 65°C)

BPTI_TPCR_R2 (reverse) CAGCTGACTTGAAGTTATTTCTCTTGGC (28 bases, 64°C)

BPTI11N2 (forward)
CAGATTTCTGCCTTGAACCACCATACNNSGGTCCCTGCAAGGCAAGGATAATTAG (26 bases, 65°C; 26 bases, 66°C)

BPTI_12N (forward) CTGCCTTGAACCACCATACACGNNSCCCTGCAAGGCAAGGATAATTAGG (22 bases, 63°C; 24 bases, 65°C)

BPTI_13N (forward) CCTTGAACCACCATACACGGGTNNSTGCAAGGCAAGGATAATTAGGTACTTTTAC (22 bases, 64°C; 30 bases 64°C)

BPTI_15N (forward) CACCATACACGGGTCCCTGCNNSGCAAGGATAATTAGGTACTTTTACAATGCG (20 bases, 64°C; 30 bases, 64°C)

BPTI_16N (forward) CCATACACGGGTCCCTGCAAGNNSAGGATAATTAGGTACTTTTACAATGCGAAAGCAG (21 bases, 66°C; 34 bases, 66°C)

BPTI_17N (forward) CGGGTCCCTGCAAGGCANNSATAATTAGGTACTTTTACAATGCGAAAGCAGG (17 bases, 65°C; 32 bases, 65°C)

BPTI18N2 (forward) GGGTCCCTGCAAGGCAAGGNNSATTAGGTACTTTTACAATGCGAAAGCAGGTC (19 bases, 66°C; 31 bases, 66°C)

BPTI34R2 (reverse) CTTGGCTCTGCAACCCCCATASNNGAAGGTCTGGCACAGACCTGC (21 bases, 65°C; 21 bases, 63°C)

BPTI_35R (reverse) CTCTTGGCTCTGCAACCCCCSNNCACGAAGGTCTGGCACAGACC (20 bases, 65°C; 21 bases, 64°C)

BPTI_36R (reverse) GAAGTTATTTCTCTTGGCTCTGCAACCSNNATACACGAAGGTCTGGCACAGACC (27 bases, 64°C; 24 bases, 64°C)

BPTI_37R (reverse) GACTTGAAGTTATTTCTCTTGGCTCTGCASNNCCCATACACGAAGGTCTGGCAC (29 bases, 66°C; 22 bases, 65°C)

BPTI39R2 (reverse) CAGCTGACTTGAAGTTATTTCTCTTGGCSNNGCAACCCCCATACACGAAGGTC (28 bases, 64°C; 22 bases, 66°C)

The Tm was calculated on this website: http://tmcalculator.neb.com/#!/. Phusion Hot Start Flex (HF Buffer), 20 nM primer concentration.
