# Supplementary material for: Generating quantitative binding landscapes through fractional binding selections combined with deep sequencing and data normalization
Source: Nat Commun. 2020 Jan 15;11:297. doi: 10.1038/s41467-019-13895-8 (PMC6962383; doi:10.1038/s41467-019-13895-8)
Supplement: Supplementary file 2 — Reporting Summary [file 41467_2019_13895_MOESM2_ESM.pdf]

nature research

Corresponding author(s): Julia Shifman, Niv papo

Last updated by author(s): Nov 17, 2019

Reporting Summary

Nature Research wishes to improve the reproducibility of the work that we publish. This form provides structure for consistency and transparency in reporting. For further information on Nature Research policies, see Authors & Referees and the Editorial Policy Checklist.

Statistics

For all statistical analyses, confirm that the following items are present in the figure legend, table legend, main text, or Methods section.

n/a	Confirmed	
☐	☒	The exact sample size (*n*) for each experimental group/condition, given as a discrete number and unit of measurement
☐	☒	A statement on whether measurements were taken from distinct samples or whether the same sample was measured repeatedly
☐	☒	The statistical test(s) used AND whether they are one- or two-sided<br>*Only common tests should be described solely by name; describe more complex techniques in the Methods section.*
☐	☒	A description of all covariates tested
☐	☒	A description of any assumptions or corrections, such as tests of normality and adjustment for multiple comparisons
☐	☒	A full description of the statistical parameters including central tendency (e.g. means) or other basic estimates (e.g. regression coefficient) AND variation (e.g. standard deviation) or associated estimates of uncertainty (e.g. confidence intervals)
☐	☒	For null hypothesis testing, the test statistic (e.g. *F*, *t*, *r*) with confidence intervals, effect sizes, degrees of freedom and *P* value noted<br>*Give P values as exact values whenever suitable.*
☒	☐	For Bayesian analysis, information on the choice of priors and Markov chain Monte Carlo settings
☒	☐	For hierarchical and complex designs, identification of the appropriate level for tests and full reporting of outcomes
☐	☒	Estimates of effect sizes (e.g. Cohen's *d*, Pearson's *r*), indicating how they were calculated

Our web collection on statistics for biologists contains articles on many of the points above.

Software and code

Policy information about availability of computer code

Data collection	BD FACSDiva v8.0.2, BD accuri C6
Data analysis	Microsoft Office Professional Plus 2013 v15.0.4569.1506, Matlab, Mathematica, Python 3.7

For manuscripts utilizing custom algorithms or software that are central to the research but not yet described in published literature, software must be made available to editors/reviewers. We strongly encourage code deposition in a community repository (e.g. GitHub). See the Nature Research guidelines for submitting code & software for further information.

Data

Policy information about availability of data

All manuscripts must include a data availability statement. This statement should provide the following information, where applicable:

- Accession codes, unique identifiers, or web links for publicly available datasets
- A list of figures that have associated raw data
- A description of any restrictions on data availability

The data that supports the findings of this study are available from the corresponding author upon reasonable request. Raw data is included in the data source file for figures 2, 3, 4, S5, S6, S7.

Field-specific reporting

Please select the one below that is the best fit for your research. If you are not sure, read the appropriate sections before making your selection.

☒ Life sciences ☐ Behavioural & social sciences ☐ Ecological, evolutionary & environmental sciences

Life sciences study design

All studies must disclose on these points even when the disclosure is negative.

Sample size	For NGS, 300,000 to 900,000 DNA strings were sequenced. The ddGbind value are reported for 228 single mutants of BPTI; each mutant was sequenced at least 100 times by NGS as described in the manuscript.
Data exclusions	As described in the paper, BPTI mutants that were sequenced less often in the pre-sorted library than the chosen cut-off, were not further analyzed. Different cut-off values were tested, their resulting sensitivity and specificity was calculated and their effects on the enrichment of synonymous mutations was analyzed.
Replication	The pre-sorted library was sequenced three times to measure the size of sequencing errors. Each binding experiment was done at least 3 times
Randomization	Since we didn't use any allocation in our study, randomization was not relevant for us.
Blinding	Since we didn't use any allocation in our study, blinding was not relevant for us.

Reporting for specific materials, systems and methods

We require information from authors about some types of materials, experimental systems and methods used in many studies. Here, indicate whether each material, system or method listed is relevant to your study. If you are not sure if a list item applies to your research, read the appropriate section before selecting a response.

Materials & experimental systems

n/a	Involved in the study
☐	☒ Antibodies
☒	☐ Eukaryotic cell lines
☒	☐ Palaeontology
☒	☐ Animals and other organisms
☒	☐ Human research participants
☒	☐ Clinical data

Methods

n/a	Involved in the study
☒	☐ ChIP-seq
☐	☒ Flow cytometry
☒	☐ MRI-based neuroimaging

Antibodies

Antibodies used	Anti-Mouse IgG (whole molecule)–R-Phycoerythrin antibody produced in goat (Sigma Aldrich, catalog number: P9287), Anti-Myc tag antibody [9E10] grown in mouse (abcam, catalog number: ab32)
Validation	Both manufacturers describe the antibodies as applicable in FACS. They were previously used in similar experiments sorting yeast cells in FACS, too (e.g. Cohen, I., et al., Biochemical Journal, 2016, https://doi.org/10.1042/BJ20151410)

Flow Cytometry

Plots

Confirm that:

☒ The axis labels state the marker and fluorochrome used (e.g. CD4-FITC).

☒ The axis scales are clearly visible. Include numbers along axes only for bottom left plot of group (a 'group' is an analysis of identical markers).

☒ All plots are contour plots with outliers or pseudocolor plots.

☒ A numerical value for number of cells or percentage (with statistics) is provided.

Methodology

Sample preparation	The yeast-displayed BPTI library was grown in SDCAA media overnight and then in SGCAA media (including galactose to induce expression). Approximately 1,000,000 to 10,000,000 yeast cells were collected, washed with PBS+1% BSA solution, incubated with 9E10 antibody for 1h at room temperature, washed again, incubated with biotinylated bovine trypsin for 1h at room temperature, washed again and then incubated for 20 min with anti-Mouse IgG-PE antibody and neutravidin (FITC).
Instrument	BD FACS AriaIII for cell sorting and collection, BD FACS accuri C6 for analysis (no sorting)

Software	BD FACSDiva v8.0.2, BD accuri C6
Cell population abundance	Sorted and pre-sorted cell populations were analyzed by NGS as decribed in more detail in the paper
Gating strategy	For FSC/SSC gates, values typical for yeast cells were used. A figure to expemplify the gating strategy is provided in the Supplementary Information.<br>To differentiate between cell populations displaying BPTI variants with different affinities, gates were chosen that have the same range in the PE signal (corresponding to BPTI expression) but different ranges in FITC signal (corresponding to BPTI binding to bovine trypsin) normalized to BPTI expression. A small gap between each gate was used to avoid BPTI variants equally enriched in two neigboring gates. To differentiate between positive and negative samples, single-stained samples and samples with no stain were used. Furthermore, a cell population only expressing BPTI-WT was used to measure its distribution of FITC and PE signal and differentiate between cells expressing BPTI variants with lower, higher or similar affinity to bovine trypsin as BPTI-WT.

☒ Tick this box to confirm that a figure exemplifying the gating strategy is provided in the Supplementary Information.
